# Procaine Inhibits Osteo/Odontogenesis through Wnt/β-Catenin Inactivation

**DOI:** 10.1371/journal.pone.0156788

**Published:** 2016-06-03

**Authors:** Carmen Herencia, Juan Miguel Diaz-Tocados, Lidia Jurado, Addy Montes de Oca, Maria Encarnación Rodríguez-Ortiz, Carmen Martín-Alonso, Julio M. Martínez-Moreno, Noemi Vergara, Mariano Rodríguez, Yolanda Almadén, Juan R. Muñoz-Castañeda

**Affiliations:** 1 Instituto Maimónides para la Investigación Biomédica de Córdoba (IMIBIC)/Hospital Universitario Reina Sofía/Universidad de Córdoba, Serv Nefrologia, Córdoba, Spain; 2 Nephrology Lab, Fundación Jiménez Díaz, REDinREN, Madrid, Spain; 3 Lipids and Atherosclerosis Unit, (CIBEROBN), Hosp Univ Reina Sofia, IMIBIC, REDinREN, Córdoba, Spain; University of Alabama at Birmingham, UNITED STATES

## Abstract

**Introduction:**

Periodontitis is a complex pathology characterized by the loss of alveolar bone. The causes and the mechanisms that promote this bone resorption still remain unknown. The knowledge of the critical regulators involved in the alteration of alveolar bone homeostasis is of great importance for developing molecular therapies. Procaine is an anesthetic drug with demethylant properties, mainly used by dentists in oral surgeries. The inhibitor role of Wnt signaling of procaine was described in vitro in colon cancer cells.

**Methods:**

In this work we evaluated the role of procaine (1 uM) in osteo/odontogenesis of rat bone marrow mesenchymal stem cells. Similarly, the mechanisms whereby procaine achieves these effects were also studied.

**Results:**

Procaine administration led to a drastic decrease of calcium content, alkaline phosphatase activity, alizarin red staining and an increase in the expression of Matrix Gla Protein. With respect to osteo/odontogenic markers, procaine decreased early and mature osteo/odontogenic markers. In parallel, procaine inhibited canonical Wnt/β-catenin pathway, observing a loss of nuclear β-catenin, a decrease in Lrp5 and Frizzled 3, a significant increase of sclerostin and Gsk3β and an increase of phosphorylated β-catenin. The combination of osteo/odontogenic stimuli and Lithium Chloride decreased mRNA expression of Gsk3β, recovered by Procaine. Furthermore it was proved that Procaine alone dose dependently increases the expression of Gsk3β and β-catenin phosphorylation. These effects of procaine were also observed on mature osteoblast. Interestingly, at this concentration of procaine no demethylant effects were observed.

**Conclusions:**

Our results demonstrated that procaine administration drastically reduced the mineralization and osteo/odontogenesis of bone marrow mesenchymal stem cells inhibiting Wnt/β-catenin pathway through the increase of Gsk3β expression and β-catenin phosphorylation.

## Introduction

Periodontitis is one of the pathologies with highest prevalence [1] and is associated with the progression of other pathologies such as Alzheimer [2], cardiovascular events, chronic kidney disease or rheumatoid arthritis [3].

It is a complex disease caused by multiple stimuli such as inflammation, diabetes, neoplasias, traumas or genetic disorders [4]. Chronic periodontitis affects tooth-supporting structures leading to periodontal ligament and bone injury and resulting in tooth loss. The causes whereby alveolar bone homeostasis is altered are currently unknown. The development of an effective treatment to reduce bone loss caused by these diseases has been a long-standing aim in dentistry [5].

One of the main stimuli for bone formation is the activation of Wnt/β-catenin pathway. Wnt signaling, which is essential for the osteogenic commitment of progenitor cells such as mesenchymal stem cells has also been shown to be activated during alveolar bone formation [6,7]. Wnt proteins are a large family of secreted molecules, that signal through binding to a co-receptor complex formed by proteins of the frizzled family and the lipoprotein receptor-related 5/6 proteins. The activation of the canonical Wnt pathway results in the nuclear translocation of β-catenin and the subsequent regulation of target gene expression and bone formation [8].

On the other hand Procaine is an anesthetic agent, mainly used for oral surgeries by dentists. It is not cytotoxic and it is approved by the FDA for local skin infiltration and topic use. In addition, procaine inhibits a group of enzymes, DNA-methyltransferases, which are necessary to catalyze DNA methylation [9,10]. Interestingly, other papers have already demonstrated that procaine may inhibit Wnt signaling through the WIF-1 promoter methylation, an endogenous inhibitor of this pathway [11]. In the context of odontology, it is interesting to know if the exposition of Procaine interferes with Wnt modulation and osteo/odontogenesis of progenitor cells. The understanding of the critical regulators associated with osteo/odontogenesis is key for developing molecular therapies for alveolar bone homeostasis and regeneration.

Therefore the main aim of this study was to investigate if the administration of procaine to progenitor cells has a negative impact on mineralization and osteo/odontogenesis of progenitor cells by blocking Wnt signaling.

## Materials and Methods

### Rat Mesenchymal Stem Cells (MSC) isolation

All experimental protocols were reviewed and approved by the Ethics Committee for Animal Research of the University of Cordoba and all rats received humane care in compliance with the Principles of Laboratory Animal Care formulated by the National Society for Medical Research.

Euthanasia was performed by aortic puncture and exsanguination under general anesthesia (50mg/kg thiopental sodium ip). Tibias and femurs of ten male Wistar rats cut at the epiphyses were perfused with alpha minimal essential medium (αMEM) medium (Sigma-Aldrich, St. Louis, MO) plus 15% fetal bovine serum (FBS) (Lonza Walkersville, Inc., USA). Following centrifugation and washing with αMEM medium, bone marrow stem cells were filtered and harvested in 25cm2 flasks (Corning Life Sciences—ALP, Chorges, France) with αMEM + 15% FBS and 1 ng/mL of basic fibroblast growth factor (bFGF) (PeproTech EC Ltd, London, UK). Fresh αMEM medium with 10% FBS and bFGF was added after 48h and successively changed 2 to 3 times per week. After reaching 90% confluence, cells were collected using Trypsin-EDTA (Lonza Walkersville, Inc., USA) and harvested in 6-well plates at 13 000 cells/cm^2^ in αMEM + bFGF. Treatments were started, as described below, when cells reached confluence.

### Treatments

Osteo/odontogenesis was induced by treating MSC with 1μM dexamethasone, 10 mM β-glycerolphosphate and 0.2 mM L-ascorbic acid (Sigma-Aldrich, St. Louis, MO) for 21 days. The effect of procaine during osteo/odontogenesis of MSC or on mature osteo/odontoblasts was studied. For the studies during osteo/odontogenesis, procaine (1 uM) (Sigma-Aldrich, St. Louis, MO) was administered at the same time that osteo/odontogenic stimulus for 21 days. For the studies on mature osteo/odontoblasts, procaine was administered for 10 days once osteo/odontoblasts were obtained for 21 days with osteo/odontogenic stimulus in MSC. Undifferentiated cells (UC) and osteoblasts like cells (OB) were treated with 10 mM lithium chloride (LiCl) (Sigma-Aldrich, St. Louis, MO), an activator of the Wnt/β-catenin pathway during 21 days in culture. In addition, MSC were cultured in presence of increasing concentrations of procaine (0, 0.5, 1 and 2 μM) for 7 days to study the effect of Procaine alone on GSK3β and phospho-β-catenin.

### RNA analysis

Total RNA was extracted with TRIzol reagent (Sigma-Aldrich) and quantified by spectrophotometry (ND-1000, Nanodrop Technologies, Wilmington, DE). Smooth Muscle protein 22 alpha (SM22α or *Tagln*), myocardin (*Myocd*), runt-related transcription factor 2 (*Runx2*), osterix (Sp7 transcription factor; *Sp7*), osteocalcin (bone gamma-carboxyglutamate (gla) protein; *Bglap*), Collagen type I (*ColA1)*, Matrix Gla Protein (*MGP*), Dentin matrix acidic phosphoprotein (*DMP1*), Receptor activator of nuclear factor kappa B ligand (RANKL), Sclerostin (SOST), Low density lipoprotein receptor-related protein 5 (*Lrp5*), Frizzled 1 and Glycogen synthase kinase 3 β (*Gsk3β*) mRNA levels were determined by quantitative real-time RT-PCR (Light cycler, Roche Diagnostics, Basel, Switzerland). cDNA was synthesized from 0.5 μg of total RNA with a first strand cDNA synthesis kit (Qiagen; Hilden, Germany) in the presence of random hexamers in a final volume of 20 μl at 25°C for 10 min, followed by 42°C for 15 min and 95°C for 3 min. An RT-PCR SYBR Green kit (Qiagen) was used to quantify mRNA expression levels. All primers, except SOST (purchased from Integrated DNA Technologies, Leuven, Belgium), were designated with the free Oligo 7 software and their sequences are listed in Table 1. mRNA expression was expressed as a value normalized to levels of 18S RNA.

**Table 1:** Primer sequences used for RT-PCR.

Gene Symbol	Gene Name	Sequence
***Myocd***	Myocardin	F:5' CTCGGAGTCAGCAGATGGATG 3´
		R:5´CCTCACTGTCGGTGGCATAGT 3´
***Tagln***	SM22α	F: 5´CACCTATCCTCAGCCTCAGC 3´
		R:5´ TCCAAAGGACATTGGCTTCC 3´
***Runx2***	Runt-related transcription factor 2	F: 5' CGGGAATGATGAGAACTACTC 3'
		R:5' GC GTCAGAGAACAAACTAGGT 3'
***Sp7***	Osterix	F:5' GTACGGCAAGGCTTCGCATCTGA 3'
		R: 5’TCAAGTGGTCGCTTCGGGTAAAG 3’
***Bglap***	Osteocalcin	F:5´ TCTGAGTCTGACAAAGCCTTCATG 3’
		R: 5’TGGGTAGGGGGCTGGGGCTCC 3’
***DMP1***	Dentin Matrix acidic Phosphoprotein 1	F: 5’GGCTGTCCTGTGCTCTCC3’
		R: 5’ACTGCTGTCCGTGTGGTC3’
***MGP***	Matrix Gla Protein	F: 5´ GCCCTGTGCTATGAATCTCACGAA 3´
		R: 5´ CGACTGTTTCCTTGCGCTCTTATT 3´
***Col1A1***	Collagen, type I	F: 5' GCAAGAACAGCGTAGCCTACATGG 3'
		R: 5' CAAGTTCCGGTGTGACTCGTGCAG 3'
***RANK-L***	Receptor Activator of Nuclear Factor kappa B Ligand	F: 5' ACGAACCTTCCATCATAGCTG 3'
		R: 5' GAAGACACAGAAGCACTACCT 3'
***MGP***	Matrix Gla Protein	F: 5´ GCCCTGTGCTATGAATCTCACGAA 3´
		R: 5´CGACTGTTTCCTTGCGCTCTTATT 3´
***Lrp5***	LDL receptor-related protein 5	F: 5’ TGTGCCACTGGTGAGATTGACT 3’
		R: 5’ ACGCTGGCAGACAAAGTAGAC 3´
***Fzd1***	Frizzled 1	F: 5' CTCTTCACGGTGCTCACGTAC 3'
		R: 5' CCAGGTGAGGGACAGGATTAC 3'
***Gsk3β***	Glycogen synthase kinase 3β	F: 5' AGCATGAAAGTTAGCAGAGAC 3'
		R: 5' TCGATTCTTAAATCTCTTGTCC 3'
**18S**	18S ribosomal RNA	F: 5´ GTAACCCGTTGAACCCCATT 3´
		R: 5´ CCATCCAATCGGTAGTAGCG 3´

### Protein extracts

Cytosolic protein was isolated from MSC in a lysis buffer containing 10 mM Hepes, 10 mM KCl, 0.1 mM EDTA, 0.1 mM EGTA, 1 mM DTT, 0.5 mM PMSF and 70 μg/mL Protease Inhibitor Cocktail, 0.5% Igepal CA-630, pH 7.9. The suspension was centrifuged and the supernatant (cytosolic extract) was stored. Protein concentration was determined by the Bradford assay (Bio-Rad Laboratories GmbH, Munich, Germany). For Western Blot, equal amounts of protein were homogenized, followed by electrophoresis in 4–20% SDS-polyacrylamide gradient gel (Bio-Rad Laboratories GmbH, Munich, Germany). The protein was transferred to a nitrocellulose membrane (Bio-Rad Laboratories GmbH, Munich, Germany), and blots were incubated in blocking solution. Primary antibodies used included goat polyclonal SM22α antibody from Novus Biologicals (Littleton, CO), (D500), rabbit monoclonal phospho β-catenin (Cell Signaling Technology, Danvers, MA), (D1:1000), rabbit monoclonal β-catenin (Cell Signaling Technology, Danvers, MA), (D1:1000) and mouse monoclonal GAPDH antibody from Santa Cruz Biotechnology (Santa Cruz, USA). Blots were immunolabeled using a horseradish peroxidase conjugated secondary antibody (D1:5000) and developed on autoradiographic film using the ECL Western Blotting Detection System from Amersham Biosciences U.K. Limited (Little Chalfont, England) in LAS 4000 (GE Healthcare Life Science, Spain).

### RunxII Transcription Factor Activity Assays

RunxII transcription factor activity was determined using a commercial TransAM^™^ assay as per manufacturer’s instructions (Active Motif, Belgium). The kits comprise a 96 well plate on which a consensus sequence for the transcription factor in question has been immobilized (RunxII, 5'-AACCACA-3'). Nuclear extracts are then added to the plates and allowed to bind. Positive controls were also included, namely nuclear extracts from cell lines transfected with constructs over-expressing RunxII. Protein bound to the consensus sequence was then treated with a specific primary antibody, which was then detected using HRP labeled secondary antibody and a colorimetric substrate. Absorbance was measured at 450 nm.

### Confocal Immunostaining

Cells were fixed with cold methanol for 20 min and subsequently washed with PBS. Fixed cells were incubated for 1 h in primary antibodies against β-catenin (BD Biosciences, CA, USA). Cells were then incubated for 1 h at room temperature with Alexa Fluor 488 anti-mouse secondary antibodies (Invitrogen Ltd, Paisley, UK). Cell nuclei were visualized with the nuclear stain 4´, 6-diamino-2-phenylindole dihydrochloride (DAPI; Invitrogen Ltd, Paisley, UK). Pictures were obtained at 40x in Axio Observer.Z1 Inverted Confocal microscope (LSM5 Exciter Zeiss).

### Alkaline Phosphatase Activity

15 μg of protein were used to colorimetrically (OD 405nm) measure alkaline phosphatase specific activity with 5 mM p-nitrophenolphosphate (Sigma-Aldrich) used as a substrate. Cell lysates were incubated in 2 mM p-nitrophenol phosphate for 30 min at 37°C. The reaction was stopped by adding 1M NaOH, and the product was quantified at 405 nm. One unit of alkaline phosphatase was defined as 1 μmol substrate hydrolyzed per hour (per μg protein/sample).

### Calcium content of cells

Cells were decalcified with 0.6 N HCl for 24 h, and the calcium content in the HCl supernatant was determined by the o-cresolphthalein complexone method (Calcium C-Test, WAKO GmbH, Neuss, Germany). Cells were washed 3 times with PBS and solubilized in 0.1mol/L NaOH 0.1% sodium dodecyl sulfate (SDS). The calcium content of the cell layer was normalized for total proteinthat was determined by the Bradford assay.

### Alizarin Red Staining

Mineral deposition was confirmed by staining with 2% (wt/vol) Alizarin Red S. The cell layers were washed twice with phosphate-buffered saline (PBS), fixed with 2% formaldehyde and 1% sucrose solution for 15 minand then washed with PBS three times. Cells were incubated in 2% alizarin red S solution pH 4.1 with shaking for 20min and subsequently washed five times with pure water pH7 and air-dried. Finally the wells were photographed.

### MTT cell proliferation assay

This test was performed at short (48 h) and long (21 days) time culture of cells with different procaine concentrations. The experiments were performed in mesenchymal stem cell (MSC) alone or in (MSC) being differentiated into osteo/odontoblasts. Cell viability was measured at 48h, 7 and 21 days after treatment with increasing concentrations of procaine (0, 0.5, 1 and 2 μM). The MTT (3-[4,5-dimethylthiazol-2-yl]-2,5-diphenyl tetrazolium bromide) (Sigma-Aldrich, St. Louis, MO) assay measures the activity of living cells via mitochondrial dehydrogenases. For this, MSC were cultured in 96 multi-well plates 13 000 cells/cm^2^ in αMEM 1 ng/mL of bFGF. The medium was changed 2 to 3 times per week. After reaching 90% confluence, cells were washed with DME without red phenol (Sigma-Aldrich, St. Louis, MO). 50 μl of MTT (1mg/ml in DME) was added to each well and incubated for another 2 hours. Then, 100 μl of isopropanol (Merck KGaA, Spain) was added to each well until the dark-blue formazan crystals formed dissolved. Finally, the absorbance was measured with a spectrophotometer at 570 nm and normalized at 650 nm. All the tests were carried out in triplicate and compared with control wells in which no procaine was added. Cell survival rate was calculated with the following equation: average absorbance value of experimental group/average absorbance value of control group × 100%.

### DNA methyltransferase activity

DNA methyltransferase (Dnmt) activity was measured by EpiQuik DNA Methyltransferase Activity/Inhibition Assay Kit (Epigentek, Brooklyn, NY, USA) using nuclear extracts from undifferentiated (UC), osteo/odontogenic cells (OB) and osteo/odontogenic cells plus procaine (OB+Procaine). Nuclear extracts were isolated with the EpiQuik Nuclear Extraction Kit (Epigentek). Dnmt activity assay was performed according to the manufacturer’s instructions. After incubation, capturing, and developing enzyme activity for samples and controls, absorbance was measured on a microplate reader at 450 nm and Dnmt activity (optical density [OD]/mg/h) was calculated according to the following formula: [(sample OD—blank OD)/(sample protein x incubation time)] x 1000.

### Statistical Analysis

Data are expressed as mean ± SE. The difference between means from two different groups was evaluated by t test; the difference between means for three or more groups was assessed by ANOVA. Statistical significance was determined according to whether the observed *p*-value was less than 0.05. Analyses were conducted using SPSS (version 15.0, Somers, NY).

## Results

### Procaine prevents osteo/odontogenic differentiation of mesenchymal stem cells

**Fig 1:**
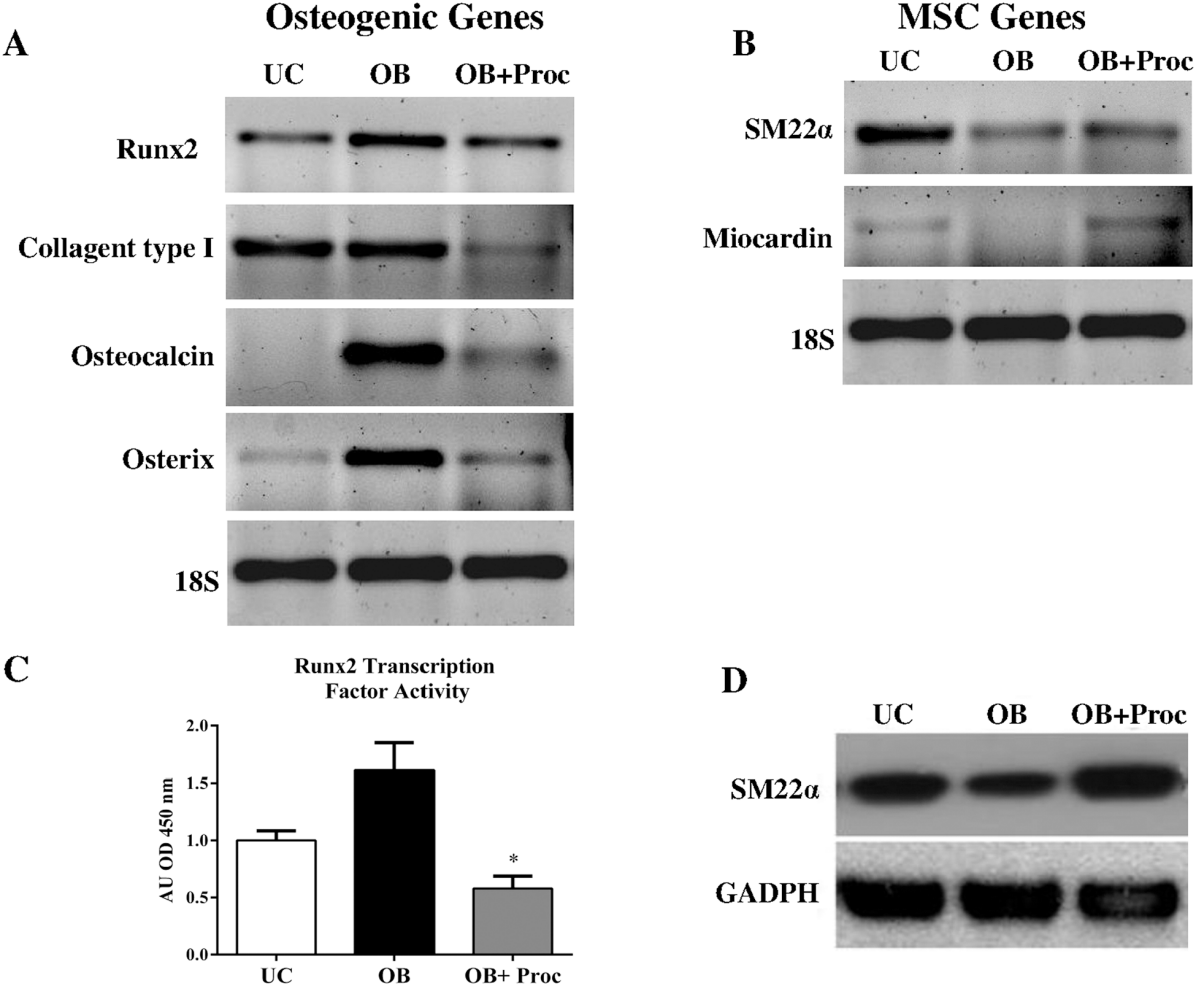
Procaine prevents osteo/odontogenic differentiation of mesenchymal stem cells (MSC). Changes in the mRNA expression of A) early markers of oste/odontogenesis and B) specific genes of mesenchymal stem cells (MSC) were determined by RT-PCR after osteo/odontogenic differentiation (OB), OB with procaine (1 μM) (OB+Proc) during 21 days. The size and intensity of amplicon was electrophoresed on agarose gel (2%). Ribosomal 18S expression was used as housekeeping. C) Runx2 transcription factor activity was determined by a commercial TransAM^™^ assay. Only OB cells showed to be significantly positive for this transcription factor (*p<0.05 vs all groups). D) Western blot for cytoplasmatic protein smooth muscle-22 alpha after 21 days of osteo/odontogenic differentiation. Images are representative of three cultures.

The efficiency of our osteo/odontogenic medium (OB) to obtain odontoblasts has been widely demonstrated [12]. Procaine administration avoided the expression of specific genes of osteo/odontoblasts. Fig 1A shows the levels of mRNA from Runx2, Osteocalcin, Osterix, Collagent type 1 of MSC differentiated into osteo/odontoblasts (OB) in presence or absence of procaine treatment for 21 days. Procaine administration (OB+Proc) significantly decreased the expression of all these markers. Fig 1B shows that during osteo/odontogenesis (OB) there is a loss of SM22α and Myocardin specific genes of MSC. On the contrary, the administration of procaine (OB+Proc) increases the expression of these markers.

At protein level, it was observed that osteo/odontogenesis (OB) increased the content of Runx2 and decreased the amount of SM22α. However the administration of procaine (OB+Proc) led to a contrary effect; decreasing Runx2 transcription factor activity and increasing SM22α protein (Fig 1C).

**Fig 2:**
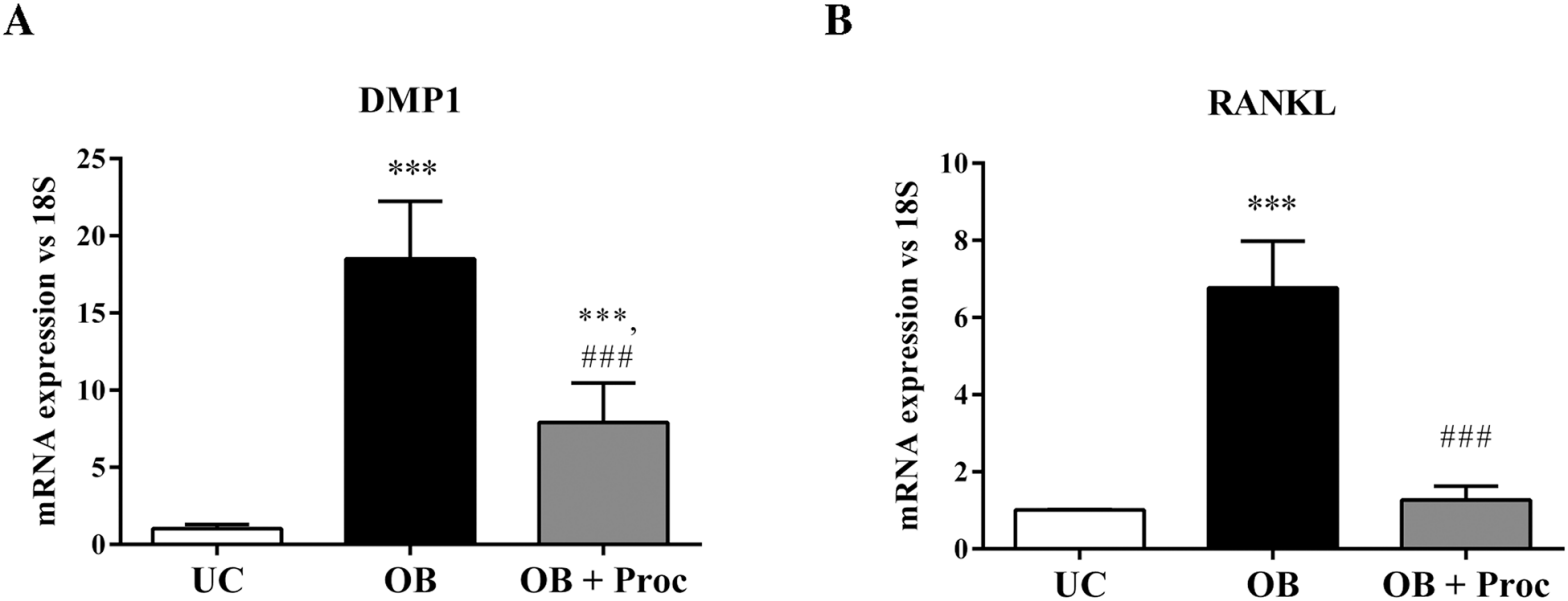
Procaine impairs osteogenic maturation of mesenchymal stem cells (MSC). mRNA expression of mature markers of oste/odontoblast such as A) DMP1 and B) RANKL were measured by RT-PCR after 21 days in culture. The expression in mesenchymal stem cell differentiated into osteo/odontoblasts (OB) was significantly higher than in undifferentiated cells (UC). This expression was reduced by the addition of Procaine (OB+Proc) during the differentiation process. Ribosomal 18S expression was used as housekeeping (* p<0.05 vs all groups).

The mRNA expression of other genes related to final maturation of odontoblasts such as Dentin Matrix acidic phosphoprotein or RANKL were modified in the same sense; increasing after osteo/odontogenic stimuli (OB) and decreasing with the procaine treatment (OB+Proc), (Fig 2).

### Procaine administration decreases mineralization during osteo/odontogenic differentiation of mesenchymal stem cells

**Fig 3:**
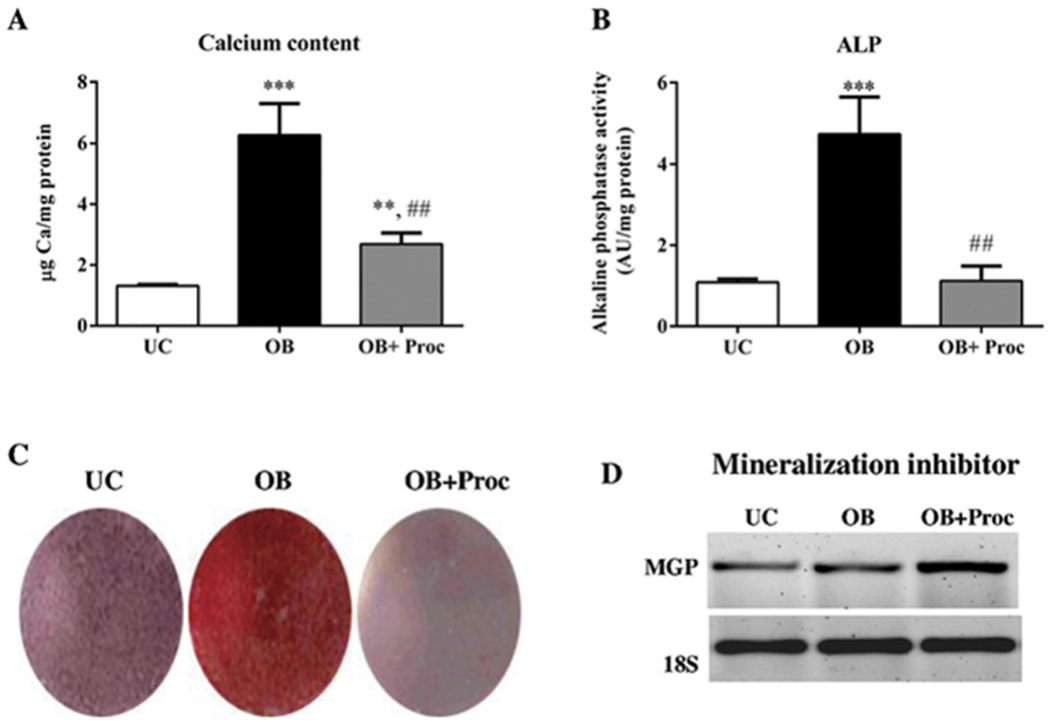
Procaine decreases mineralization of osteo/odontoblasts differentiated from mesenchymal stem cells (MSC). A) Calcium content and B) alkaline phosphatase activity were significantly increased after osteo/odontogenic stimulus (OB) (* p< 0.05 vs. undifferentiated cells (UC)) at 21 days. Procaine addition (OB+Proc) significantly decreased calcium content and alkaline phosphatase activity after 21 days of differentiation (• p< 0.05 vs. OB cells). C) Alizarin red staining increased after osteo/odontoblasts differentiation (OB) while procaine addition (OB+Proc) significantly decreased mineralization during the differentiation process. Images are representative of three cultures. D) MGP gene expression was increased after procaine administration, impairing the mineralization process in OB cells. The size and intensity of amplicon was analyzed on agarose gel (2%). Ribosomal 18S expression was used as housekeeping.

To evaluate the effects of procaine on mineralization during osteo/odontogenesis of MSC the levels of calcium, alkaline phosphatase activity, Alizarin Red Staining and MGP expression were evaluated. Fig 3 shows that osteo/odontogenic stimulus (OB) leads to increases in calcium content (Fig 3A), ALP activity (Fig 3B), Alizarin Red staining (Fig 3C) and a decrease of MGP expression (Fig 3D). The administration of procaine (OB+Proc) inhibited all the parameters related to mineralization.

### Procaine inhibits Wnt/β-catenin avoiding nuclear translocation of β-catenin

**Fig 4:**
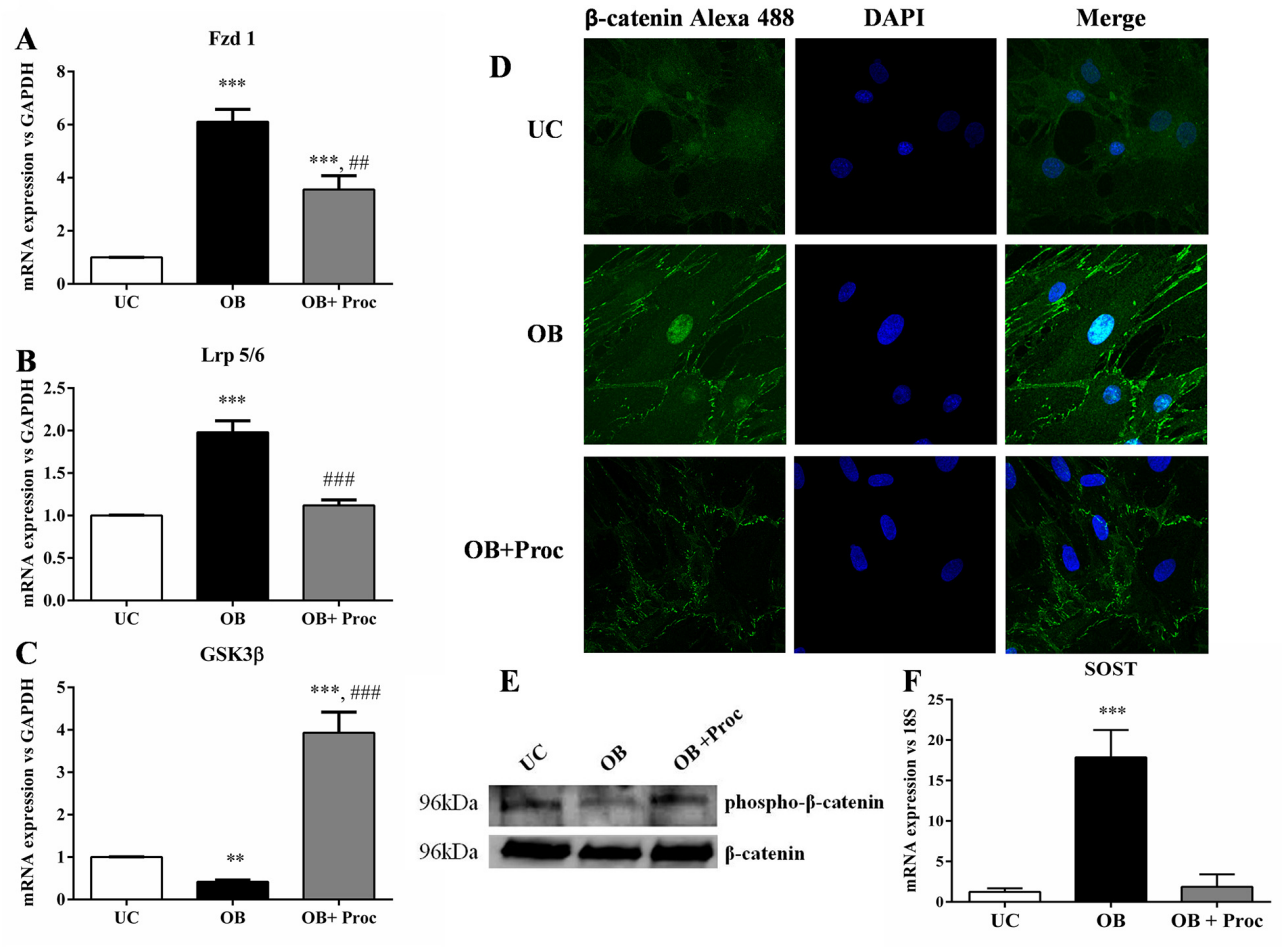
Procaine down-regulates Wnt/β- catenin pathway during osteo/odontogenesis of MSC. Osteo/odontoblast stimulus (OB) increased the mRNA expression of A) Lrp5/6 and B) Frizzled 1 and decreased the mRNA expression of C) Gsk3β (***p<0.05 vs. undifferentiated cells, UC. Treatments with procaine (OB+Proc) decreased expression of Lrp5/6 and Frizzled 1 and increased expression of Gsk3β (### p<0.001 vs OB cells and ## p<0.01 vs OB cells). D) Rat mesenchymal stem cells treated with osteo/odontoblasts stimulus (OB) or osteo/odontoblasts stimulus plus procaine (OB+Proc) were stained for β-catenin immunofluorescence (green) and counterstained with DAPI (blue) to determine β-catenin subcellular localization. Merged images of β-catenin immunofluorescence and DAPI staining are shown. Original magnification: 40x. Images were representatives of three independent experiments. E) phospho-β-catenin analysis by western blot showed that osteo/odontogenesis of MSC leads to a decrease of this protein while the presence of Procaine recovered the cytoplasmic levels of phospho β-catenin after 21 days of culture. Images are representative of three cultures. F) mRNA expression of sclerostin was decreased by osteo/odontoblast stimulus (OB) (p<0.05 vs UC) while procaine administration significantly increased the expression of this inhibitor (p<0.05 vs OB). Ribosomal 18S expression was used as housekeeping.

The expression of Wnt/β-catenin related genes such as Frizzled 1, Lrp5/6, GSK3β or SOST was also analyzed. Osteo/odontogenic stimuli (OB) led to the activation of Wnt/β-catenin pathway through the increase of Frizled1 or Lrp5/6 (Fig 4A and 4B) and the decrease of GSK3β expression (Fig 4C). Confocal analysis detected the presence of nuclear β-catenin at 14 days in MSC treated with osteo/odontogenic stimulus (OB). The procaine treatment (OB+Proc)avoided the Wnt/β-catenin activation through the decrease in the expression of mRNA expression of Frizzled 1 (Fig 4A), Lrp5/6 (Fig 4B) and the increase of GSK3β (Fig 4C). Furthermore, with procaine there was an intense presence of β-catenin in the cell membrane so nuclear translocation of this protein was avoided (Fig 4D). Osteo/odontogenic differentiation is associated with a decrease of phospho-β-catenin compared to undifferentiated cells (UC) and the administration of procaine (OB+Proc) promoted the phosphorylation of β-catenin in Tyr residues (Fig 4E). Finally it is interesting to note that procaine (OB + Proc) also increased the expression of sclerostin, endogenous inhibitor of canonical Wnt/β-catenin pathway, which increased significantly (Fig 4F), with respect to osteo/odontoblast cells (OB).

### Mechanisms of action of Procaine

**Fig 5:**
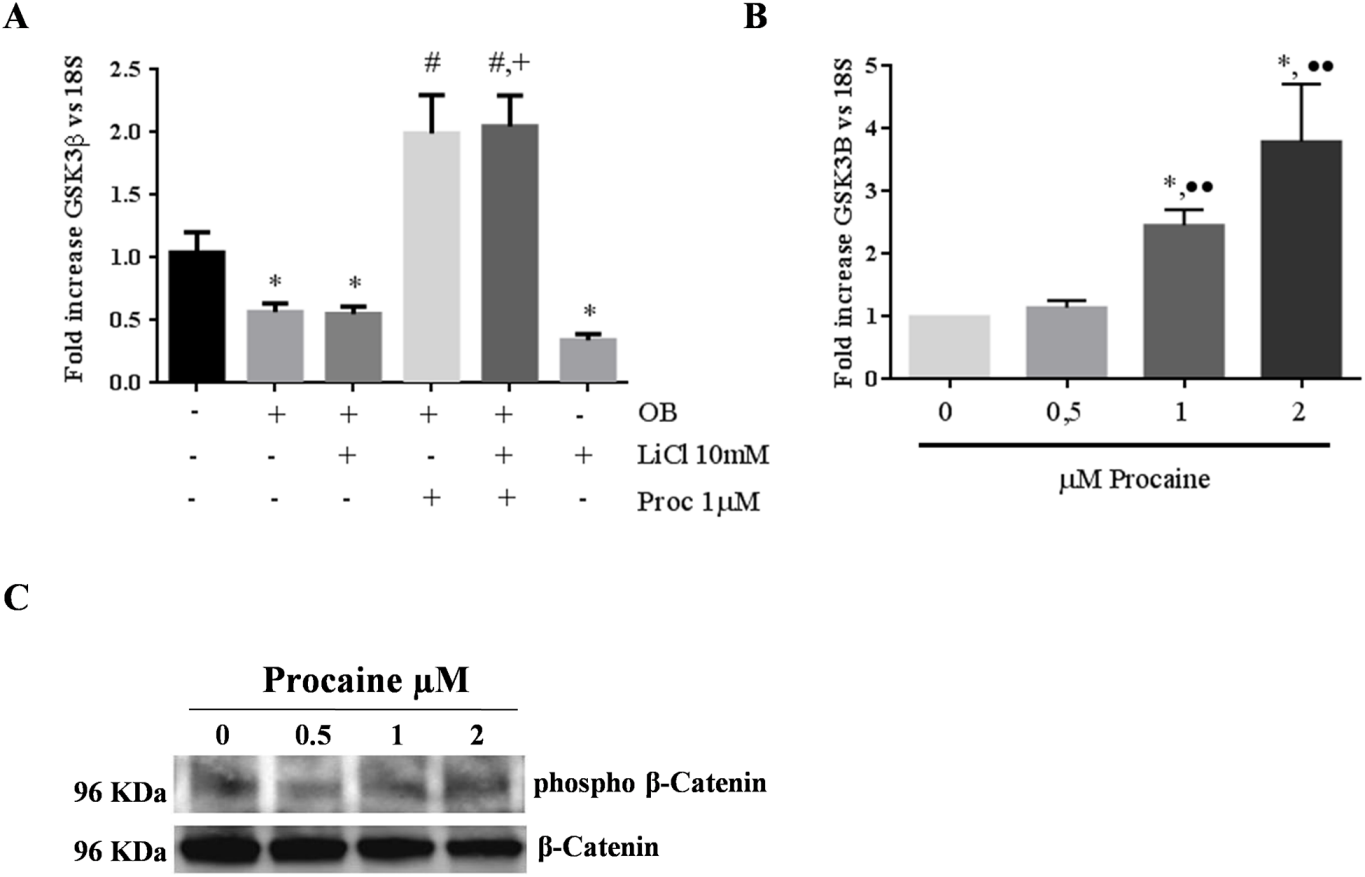
Mechanism of action of procaine. Changes in mRNA expression of GSK3β from undifferentiated mesenchymal stem cells (UC), MSC differentiated into osteo/odontoblast (OB), MSC differentiated into osteo/odontoblast plus procaine (1μM) (OB+Proc), MSC differentiated into osteo/odontoblast plus lithium chloride (10 mM) (OB+LiCl) and the combination of osteo/odontogenic stimuli, Procaine (1μM) and Lithium Chloride (10 mM) were analyzed A). Effect of increasing concentrations of Procaine (0, 0.5, 1 and 2 μM) on GSK3βexpression B) and phospho-β-catenin C) for 7 days on MSC. * p<0.05 vs UC; # p<0.05 vs OB; + p<0.01 vs OB+LiCl; ••p<0.01 vs. 0.5 μM Proc.

During MSC differentiation into osteo/odontoblasts there was a significant down-regulation of GSK3βmRNA. This effect was similar to that observed with LiCl (10 mM) alone or in combination with the stimuli used for osteo/odontoblast differentiation. The addition of procaine plus LiCl to MSC differentiated into osteo/odontoblasts caused elevation of GSK3β expression (Fig 5A).

In MSC maintained in culture for 7 days, increasing concentrations of procaine (0, 0.5, 1 and 2μM) produced a concentration dependent up-regulation of GSK3βm RNA(Fig 5A). This action waschecked by analysing the levels of cytoplasmic phospho β-catenin by western blot. These levels increased in parallel to procaine concentration. Cytoplasmic unphosphorylated β-catenin was used as loading control (Fig 5C).

### Non cytotoxic effect of Procaine

**Fig 6:**
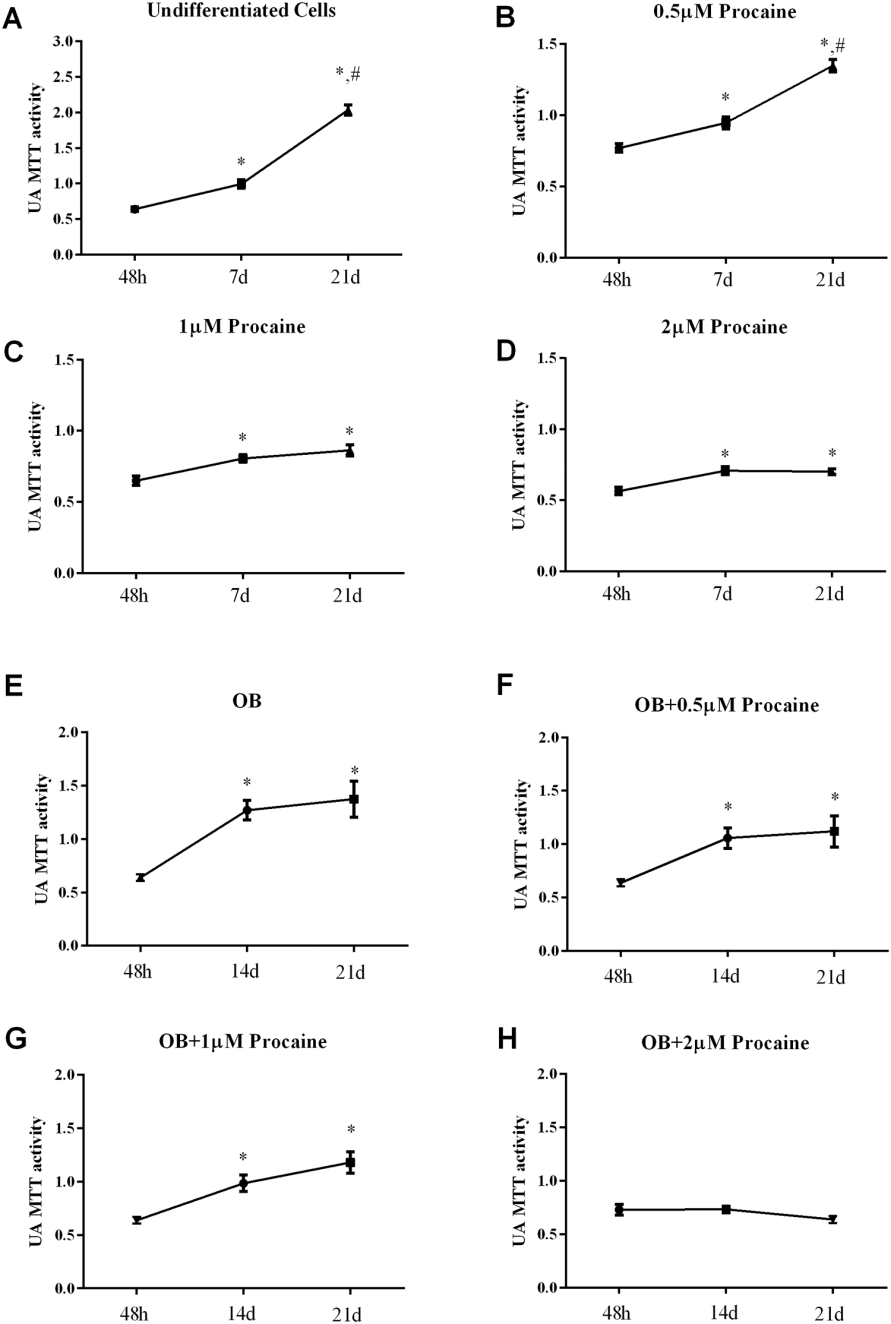
Non cytotoxic effect of Procaine. The effect of several doses of procaine (0.5, 1 and 2 μM) on cell proliferation and viability was studied onundifferentiated mesenchymal stem cells (UC) (A), MSC plus Procaine 0.5 μM (B), 1 μM (C) y 2 μM (D) for 48h, 7 and 21 days of treatment. Changes in cell proliferation and viability were also studied on MSC differentiation into osteo/odontoblasts (E) at 48h, 14d and 21d. The effects of procaine on cell proliferation and viability on these osteo/odontoblasts were studied at 0.5 μM (F), 1 μM (G) and 2 μM (H). * p<0.001 vs 48h and # p<0.001 vs 7d.

MTT assay was performed to analyze a potential toxic effect of procaine alone (Fig 6A–6D) or plus osteo/odontogenic stimuli (OB) (Fig 6E–6H). In comparison with a short incubation period (48 h), neither treatment with procaine reduced MTT activity after 7 or 21 days. An increase in proliferation was observed in undifferentiated cells with 0.5 μM of procaine. With 1 and 2 μM of procaine there was no decrease in cell viability as compared with 48h culture (Fig 6A–6D). This effect was similar during MSC differentiation into osteo/odontoblasts. Different doses of Procaine (0.5, 1 and 2 μM) did not produce less proliferation or toxic effects in comparison to its corresponding initial time of 48h or 14 days (Fig 6E–6H).

### Effects of procaine on mature osteoblasts

**Fig 7:**
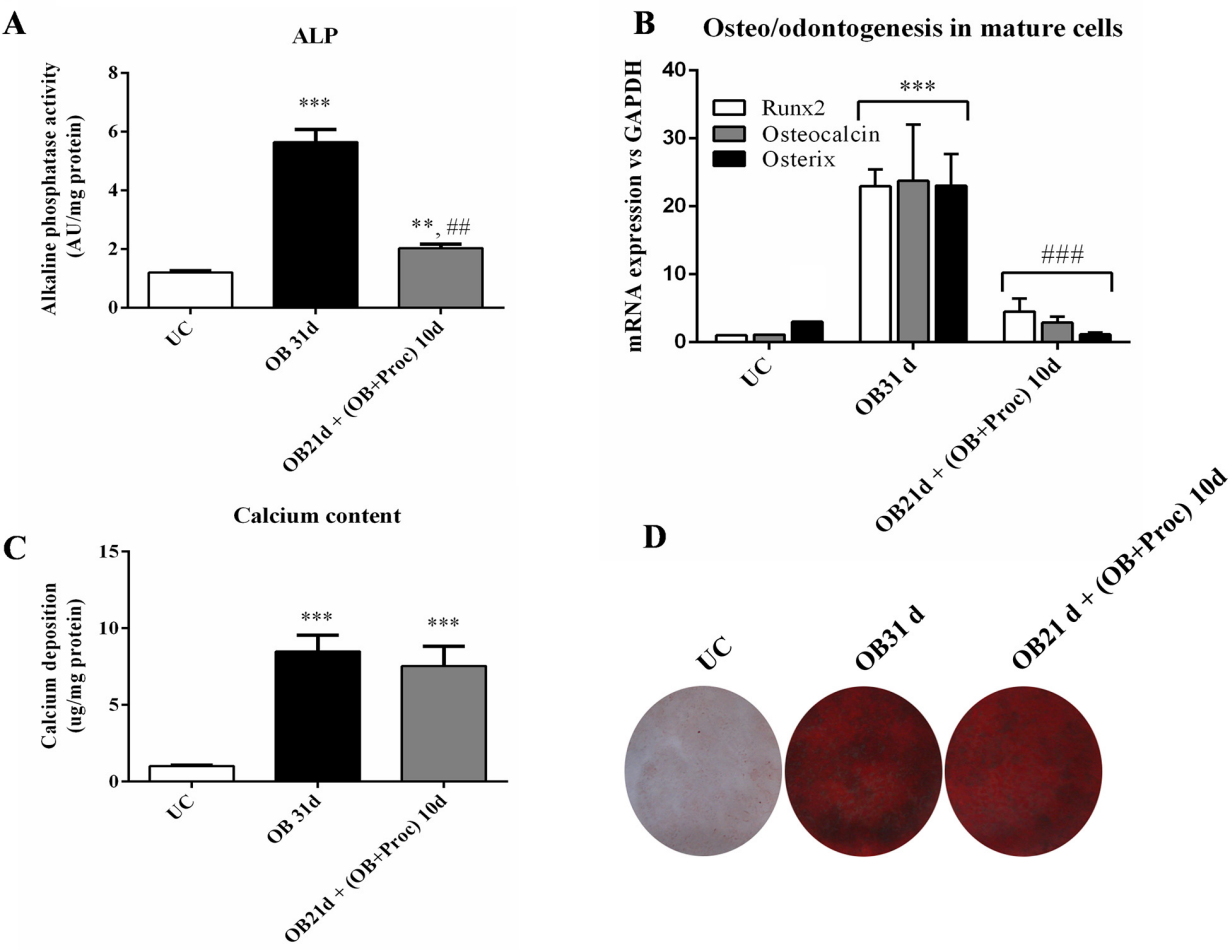
Procaine also promotes changes on mature osteo/odontoblasts. Procaine addition for 10 days to differentiated osteoblasts cells from MSC for 31days led to a decrease in A) alkaline phosphatase activity (*** p<0.001, ** p<0.01 vs. undifferentiated cells (UC) and ## p<0.01 vs OB cells) and B) on osteo/odontogenic genes (***p<0.001 vs UC cells and ### p<0.001 vs. OB cells). C) Calcium content and D) alizarin red staining did not change with respect to OB cells after 31 days of osteo/odontogenic stimulus and the last 10 days with procaine. Images representative of three experiments.

Additional experiments adding procaine for 10 days on mature osteo/odontoblasts were carried out to check the effects of this drug on mature osteoblasts. We observed that procaine administration (OB21 d + (OB+Proc) 10d) induced a decrease of alkaline phosphatase activity (Fig 7A) and of the expression of osteogenic markers such as Runx2, Osterix and Osteocalcin (Fig 7B). However there were no differences in calcium levels or Alizarin Red staining (Fig 7C and 7D).

### Effects of procaine on DNA methylation

In order to analyze the demethylant effect of procaine, we measured the global activity of methyltransferases enzymes. Our results showed that during osteo/odontogenic differentiation (OB) there was a significant increase in the activity of methyltransferase enzymes. The treatment with procaine (OB+Proc)at this concentration did not modify the activity of these enzymes with respect to the osteo/odontogenic group (S1 Fig).

## Discussion

The present study shows that procaine decreases both osteo/odontogenesis and mineralization of bone marrow MSC differentiated into osteo/odontoblasts. This effect is associated with an inhibition of nuclear translocation of β-catenin and Wnt/β-catenin down-regulation. In this study, a demethylant effect of procaine was not observed. In comparison with other studies with this drug, we detect significant differences regarding findings with high doses of procaine (between 1–10 mM). In our work, a final concentration of 1μM of procaine was added and this may be the reason why we did not observe changes in DNA methyltransferase enzymes (S1 Fig). However, a powerful action of procaine on Wnt signaling was observed. Our study evaluates, for the first time, the relationship between procaine, osteo/odontogenesis and Wnt signaling in bone marrow MSC. Procaine inhibited osteo/odontoblast mineralization of MSC decreasing calcium content, alkaline phosphatase activity, alizarin red staining and increasing MGP expression. It is interesting to note that during osteoblasts differentiation a decrease in MGP expression was produced while procaine treatment notably increased the expression of this inhibitor of mineralization. Kaipatur NR et al, using an overexpression of MGP through osteoblast/odontoblast-specific 2.3-kb Col1a1 promoter, demonstrated that there was a decrease in the mineralization of tooth root dentin and cellular cementum, while crown dentin showed "breakthrough" areas of mineralization [13]. These results suggest that bone and tooth mineralization is critically regulated by mineralization inhibitors. Other authors have shown that MGP-deficient mice exhibited extensive artery calcification [14] and MGP deficiency in mice aortas induced a diffuse calcification of vascular medial cells [15].

The action of procaine in the context of mineralization and osteo/odontogenesis is unknown. In planulae and polyps of the hydroid Hydractinia symbiolongicarpus,procaine administration reduced the calcification by calcium carbonate [16]. Data published by our group has shown that procaine inhibits calcification of VSMC through a demethylation of SM22α promoter [17].

In parallel, procaine achieved to decrease both the expression of early markers of osteo/odontogenesis (RunxII, Osterix, Osteocalcin, Collagen typeII) and mature markers (DMP1, RANKL or SOST). It is known that during osteo/odontogenic differentiation of bone marrow MSC, β-catenin is translocated into nuclei activating canonical Wnt signaling and promoting or inhibiting the expression of specific genes to achieve osteo/odontoblast differentiation [18–20]. Our results showed that during osteo/odontoblast stimuli the Wnt/β-catenin pathway was activated. This was illustrated by the nuclear translocation of β-catenin, the over-expression of Frizzled 1 and Lrp5/6 and the down-regulation of GSK3-β. Activation of Frizzled 1 and LRP5/6 co-receptors leads to inhibition of GSK3-β activity followed by the accumulation and nuclear translocation of unphosphorylated β-catenin. Similar results have been observed by others [19, 21]. Shan T et al observed that the Wnt pathway activator LiCl can promote proliferation and odontoblast differentiation of hair follicle neural crest cells, increasing the expression of some odontoblast markers such as DMP1 or Runx2 [22]. It is interesting to note that although SOST is produced mainly in mature osteoblasts, the administration of procaine promoted a significant increase of this inhibitor of canonical Wnt signaling. This increase might be responsible for the Wnt pathway inhibition and the observed delay in osteo/odontogenesis of progenitor cells. Sclerostin is expressed by osteocytes and cementocytes [23]; however, its role in the formation of dental structures remains unclear. The role of Wnt/β-catenin results particularly important in the context of periodontitis. It was documented that SOST and DKK1 were upregulated in the periodontal tissues of chronic periodontitis subjects, suggesting a possible role of these molecules on periodontal tissues [24]. Finally, it was also demonstrated that pharmacologic inhibition of SOST, using a SOST neutralizing monoclonal antibody, restored the alveolar bone destruction following experimental periodontitis in rats [25]. The removal of Wnt activity in the periodontium results in major defects in alveolar bone formation and periodontal ligament [26]. Enhancing Wnt activity by removing its antagonist, sclerostin, leads to an increase in alveolar bone volume and reduced periodontal ligament width [23]. Changes in Wnt signaling are the most likely events that alter the differentiation of progenitor cells and alveolar bone formation, leading to bone loss.

With the increase in GSK3β expression, we observed a parallel increase in cytoplasmic phosphorylated β-catenin and a reduction of unphosphorylated β-catenin in the nucleus. These results suggest that procaine reduces differentiation to osteo/odontogenesis by increasing GSK3β and β-catenin phosphorylation with the subsequent potential degradation in the proteasome.

Procaine inhibits LiCl induced dephosphorylation promoting GSK3 β expression and Wnt inactivation. This finding is of interest in the interpretation of those pathologies where a canonical Wnt/β-catenin pathway is activated aberrantly, such as some tumoral processes.

Our data reveal that the addition of procaine inhibits cell proliferation without producing cell toxicity in undifferentiated MSC or in MSC differentiated into osteo/odontoblasts. This data goes alone with the known effect of inhibition of canonical Wnt signaling by procaine, which decreases cell proliferation.

The anti-osteo/odontogenic effects of procaine were also observed on mature cells. This finding illustrates the powerful anti-osteogenic effect of procaine. It is tempting to propose this molecule as an efficient inhibitor of Wnt signaling. However, the required plasmatic concentration to trigger in vivo effects is yet unknown. Others have observed the effects of procaine on Wnt signaling. Gao et al. showed that procaine inhibits Wnt/β-catenin through a promoter demethylation of WIF-1 in lung cancer cells [11] however the potential interaction of procaine and the Wnt/β-catenin pathway in the context of osteo/odontogenesis was unknown.

Despite the absence of in vivo studies or clinical trials analyzing the role of procaine as a direct inhibitor of canonical Wnt signaling, it would be interesting to test the efficacy of this drug in in vivo studies where nuclear translocation of β-catenin was responsible for the generation of pathological process such as some types of hepatocarcinome or colon cancers.

It is unknown if in patients with chronic periodontitis and loss of alveolar bone, an excessive use of procaine might contribute to inhibit Wnt signaling, reducing bone formation and even promoting a loss in the phenotype of cells that compound alveolar bone.

In conclusion, our data show a clear anti-osteo/odontogenic effect of procaine on the mineralization and differentiation of rat MSC into osteo/odontoblasts. This effect is mediated through the increase of GSKβ expression and the phosphorylation of cytoplasmic β-catenin inhibiting nuclear translocation of β-catenin and this canonical pathway. These effects at this dose are independent of changes in DNA-methylation.

Finally more studies with similar drugs such as lidocaine should be performed to analyze their effect on osteo/odontogenesis and Wnt signaling.

## Supporting Information

S1 Fig Procaine does not induce changes in the activity of DNA methyltransferase enzymes. Osteo/odontogenic stimuli (OB) increased significantly the activity of DNA methyltransferase enzymes. Procaine administration at 1 μM (OB+Proc) does not modify the activity of these enzymes with respect to OB and resulted to be significantly higher than UC (***p<0.001 vs undifferentiated cells (UC).
(TIF)
