# Supplementary figures and images for: Procaine Inhibits Osteo/Odontogenesis through Wnt/β-Catenin Inactivation
Source: PLoS One. 2016 Jun 3;11(6):e0156788. doi: 10.1371/journal.pone.0156788 (PMC4892678; doi:10.1371/journal.pone.0156788)

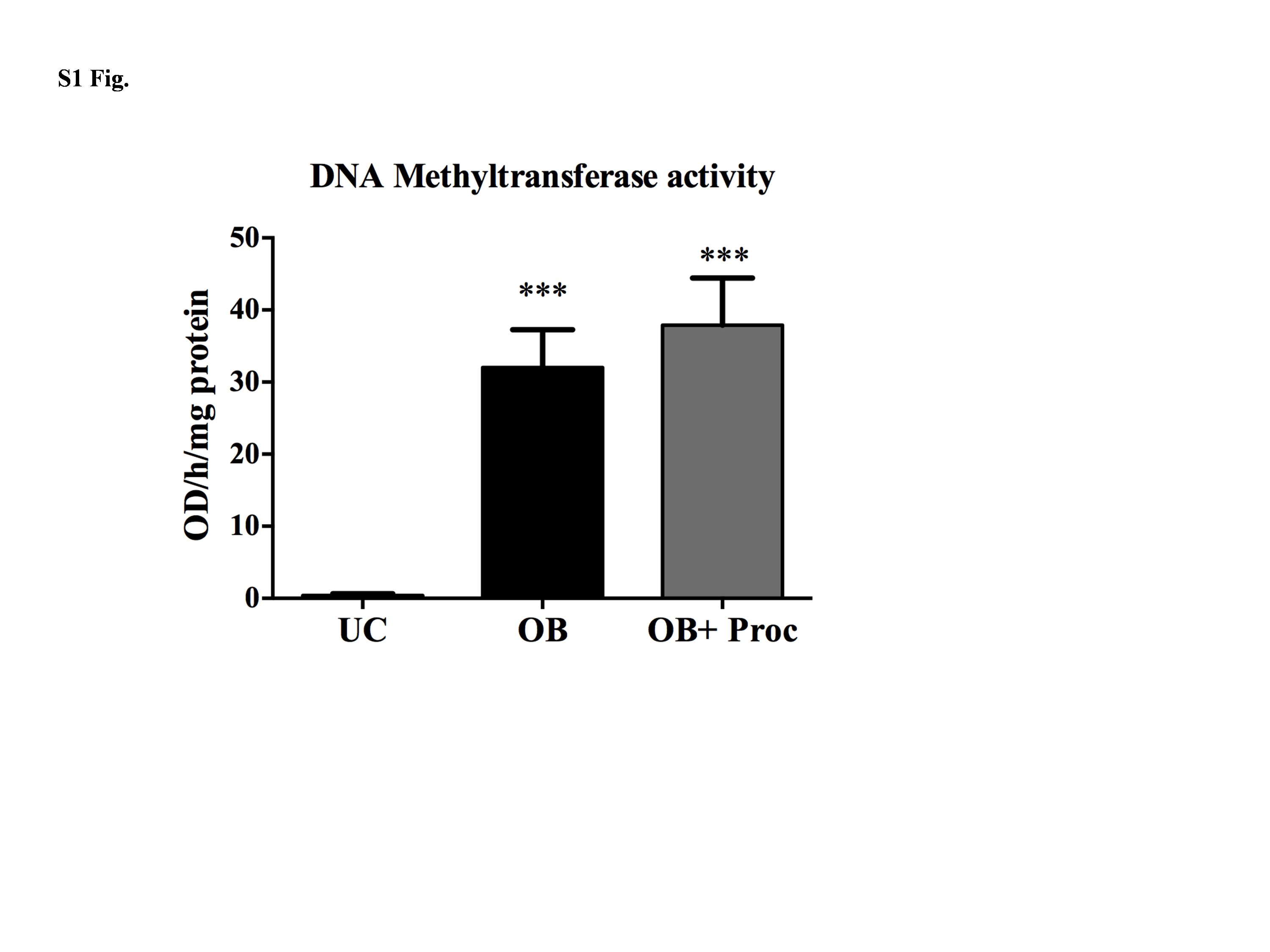

Supplement: S1 Fig — Osteo/odontogenic stimuli (OB) increased significantly the activity of DNA methyltransferase enzymes. Procaine administration at 1 μM (OB+Proc) does not modify the activity of these enzymes with respect to OB and resulted to be significantly higher than UC (***p<0.001 vs undifferentiated cells (UC). (TIF) [file pone.0156788.s001.tif]
